# Cellular and Molecular Level Mechanisms against Electrochemical Cancer Therapy

**DOI:** 10.1155/2019/3431674

**Published:** 2019-04-14

**Authors:** Yeun-Hwa Gu, Takenori Yamashita, Tota Inoue, Jin-Ho Song, Ki-Mun Kang

**Affiliations:** ^1^Department of Radiological Science, Faculty of Health Science, Junshin Gakuen University, 1-1-1 Chikushigaoka, Minami-ku, Fukuoka 815-8510, Japan; ^2^Department of Radiological Science, Faculty of Health Science, Suzuka University of Medical Science, 1001-1, Kishioka, Suzuka City, Mie 510-0293, Japan; ^3^Mie Breathing Swallowing Rehabilitation Clinic, 14-7 Iris Town-cho, Kameyama City, Mie 519-0071, Japan; ^4^Department of Radiation Oncology, Gyeongsang National University School of Medicine and Gyeongsang National University Changwon Hospital, 11 Sangjeongja-ro, Seongsan-gu, Changwon City, Gyeongsangnam-do, Changwon 642120, Republic of Korea

## Abstract

Electrochemical treatment (ECT) is a promising new way to induce tumor regression by flowing direct current into the cancer tissue. ECT was applied to different kinds of tumors in clinical studies and showed good results. In addition, basic research has almost not been done in the field of evaluation of efficacy, dose-response, and cytotoxicity. Therefore, the objective is to study the cellular mechanism in the antitumor effect of ECT and to contribute data of basic research of ECT. In the cell-level study, tumor cells (Sarcoma-180, Scc-7, Ehrlich Carcinoma) were studied using ICR mice and C3H mice. In the study group, pH values of control, 10mA × 150secs, 10mA × 300secs, and 10mA × 600secs groups were measured five times each. In histological level studies, ECT was performed on tumors inoculated on the upper part of the right foot of C3H mice. In each group, mice were sacrificed by cervical dislocation 6, 12, and 24 hrs after ECT treatment, and tumors were removed. The excised tumor was fixed in tissue with 10% formalin, and HE staining and apoptosis antibody staining were carried out from the obtained tissue section and observation. In the study at the cellular level, statistically significant differences were observed in all ECT groups in Sarcoma in the tumor growth measurement study compared with the control group. Statistically significant differences were also observed in Scc-7 in all ECT groups compared to the control group. In the intratumoral pH measurement study, there was a statistically significant difference between the anode and the cathode in each group compared to the control group. In the examination at the histological level, microscopic observation of a slide stained with apoptosis antibody with a magnification of 400 times showed that 6hrs after ECT it was stronger and then decreased. By performing ECT, a weak current flows in the living body. As a result, changes in tissue pH, generation of gas, etc. occur. In this study, it was also confirmed that the intratumor pH value becomes strongly acidic on the anode side and strongly alkaline on the cathode side. In addition, this study confirmed the occurrence of gas during treatment of ECT. Changes in the pH and the like cause changes in the environment in the cell, denaturation of proteins, apoptosis, and necrosis. In this study, a significant increase in apoptosis was confirmed in each ECT group compared to the control group. Treatment effects by ECT were also observed in tumor growth measurement studies and tumor weight measurement studies. From these research results, ECT is considered to be effective as a tumor treatment method.

## 1. Introduction

At present, three major therapies (surgical therapy, radiotherapy, and chemotherapy) are the mainstream of cancer therapy. Surgical therapy is a method of surgically removing lesions or tumor cells and normal tissues and can be treated completely only when the cancer remains in the original lesion. Radiotherapy is a local treatment that treats only cancer and surroundings like surgical therapy, but unlike surgical therapy it is a treatment method that does not have to remove the organ. Currently used radiation includes X-rays, *γ*-rays, proton beams, heavy particle beams, and the like [1].

Chemotherapy is a treatment method that uses chemical substances to suppress the division of cancer and kill cancer cells. The anticancer drug enters the blood after administration and destroys the cancer cells surrounding the whole body, so it has a systemic effect. Cancer is called systemic disease, and in the early stage local focal lesion that is limited to a certain site gradually transfers to the whole body and becomes a systemic illness. Surgical therapy and radiotherapy are used to treat local cancers, but chemotherapy is considered to be a more appropriate treatment for cure of systemic diseases. However, side effects such as physical burden and decline in immunity cannot be avoided commonly in the three major treatments. Recent improvements in the quality of life (QOL) of patients are considered, and therapeutic methods with fewer side effects such as immunotherapy have been drawing attention. Currently, these treatments are used alone or in combination [1].

ECT is a promising new method of inducing tumor regression by flowing direct current into the cancer tissue [2–4]. Nodenstrom first used direct current to treat human lung tumors [5, 6]. Growing fetuses and proliferating tumors are electrically negative to the abdominal wall outside and are more negative than tissue with slower proliferation rate or stagnation. Various studies have been carried out since Humphrey in 1958 reported the disappearance of Sarcoma-180 tumor in rats by direct current application in 1958, considering that they could supply electricity from outside and stop tissue growth and proliferation [7, 8]. Xin et al. reported that ECT was performed on more than 6000 different tumors by 1994, and overall the total response (CR) and partial response (PR) were 71.8% [6].

ECT was applied to different kinds of tumors in clinical studies and showed good results, but almost no organizational study was done in this field.

In addition, fundamental research has not been done in the field of efficacy, dose-response, cytotoxicity evaluation, and the biological mechanism of ECT has not been determined yet. Therefore, in our research, the purpose of the research is to establish ECT mechanism and optimum conditions by using mice. It is aimed to study mechanisms of cellular level and DNA level in the antitumor effect of ECT and to contribute data to fundamental research of ECT.

## 2. Methods

**Figure 1:**
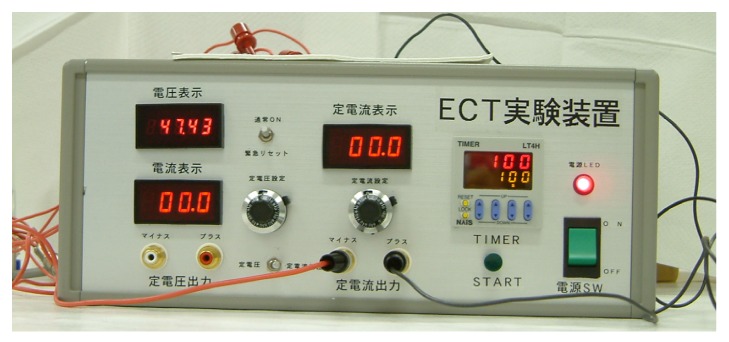
Electrochemical therapy equipment (Sunny Health Co, Tokyo, Japan).

Figure 1 shows the ECT equipment used this time.

pH measuring instrument: purchased from HORIBA (Model: D-21, Model: EX-20, Power: 9 V DC).

pH electrode: purchased from Fuji Chemicals Co., Ltd. Glass Electrode (special order).

### 2.1. Studies in the Cellular Level

Closed Coloney's ICR [Crj: CD-1] mouse and inbred C3H/HeNCrj mouse were used at Japan SLC. In order to familiarize themselves with the breeding conditions of this study, we purchased preliminary breeding for one week and used it for research. Rearing conditions were bred under Conventional conditions. The room temperature was 22 ± 3°C and the humidity was 60 to 70%.

In the light control, water and bait (CA-1, Japan CLEA Co., Ltd.) freely ingested the light period of 12 hours from 6:00 A.M. to 6:00 P.M. As a comparative control group, the control group with only tumor inoculation and the antitumor effect group were grouped into 3 groups: tumor + 10mA × 150sec group, tumor + 10mA × 300sec group, and tumor + 10mA × 600sec group. In this study, Male mice of 6 weeks of age in each group were used in each group.

### 2.2. Tumor Cell

Sarcoma-180 tumor cells are mouse sarcoma cell line.

Ehrlich-Lettre ascites carcinoma (EAC) is also known as Ehrlich cell. It was originally established as an ascites tumor in mice.

The other cell lines were supplied by the Cell Resource Center for Biomedical Research, Tohoku University (Sendai, Japan) and Riken Cell Bank (Tsukuba, Japan), respectively.

The FSA fibro sarcoma (following Sarcoma) and Ehrlich carcinoma (following Ehrlich) were inoculated into ICR mice and the SCC-7 squamous cell carcinoma (following Scc-7) was inoculated into C3H mice for research.

Sarcoma and Ehrlich pulled from the mouse intraperitoneally by withdrawing from the mouse and washed the cells several times, so that the cells became about 1× 10^6^ cells in 0.05 ml of 0.9% saline and used for research. There was Scc-7 stored in liquid nitrogen, thawed, and warmed in an incubator with sufficient humidity under 5% carbon dioxide, mixed with Fetal Bovine Serum in RPMI 1640, and kept in incubator for 2 and 3 months. Approximately 1 × 10^6^ cells were poured from the mixed solution and mixed with 0.05 ml serum free medium and used for research. The inoculation sites of Sarcoma, Ehrlich, and Scc-7 were inoculated subcutaneously in the upper right part of the mouse's right foot.

### 2.3. Anesthesia

Pentobarbital sodium was purchased from Dainippon Pharmaceutical. It was formulated with 0.9% physiological saline to a concentration of 10%, administered intraperitoneally, and used for anesthesia. The dose concentration was 10ml/kg of 10% Pentobarbital sodium. The study was initiated approximately 10 minutes after peritoneal administration and was used during ECT study.

### 2.4. ECT Treatment Method/Condition

**Figure 2:**
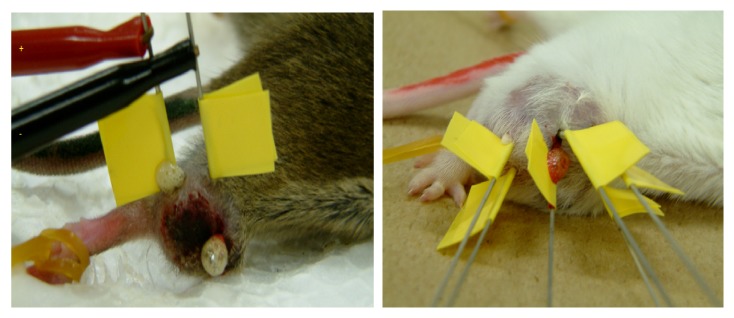
Case of electrochemical therapy in the right upper thigh mass. There is 1cm distance between the electrodes.

ECT was performed when the long and short diameter of the tumor inoculated on the upper part of the mouse's right foot reached the size of 2cm × 2cm in Sarcoma (Figure 2).

In Ehrlich and Scc-7, ECT was performed when the major axis and minor axis reached 10mA × 150secs × 10mA × 15secs. As treatment conditions, the electrode was inserted throughout the tumor so that the distance between the anode and the cathode was within 1 cm, and ECT was performed under Pentobarbital sodium anesthesia with the currently used 10mA. Wounds were carefully disinfected with 70% ethanol before and after the insertion of the electrode.

### 2.5. Tumor Growth Measurement Study

ECT was performed when the major axis and the minor axis of the tumor inoculated on the upper part of the mouse's right foot reached a size of 2cm × 2cm for Sarcoma. Thereafter, in Ehrlich, Scc-7, ECT was performed when the major axis and minor axis became 1cm × 1cm in size, and the growth retardation state of the tumor was observed every day until the 14th day, and the effect of ECT was judged.

The volume of the tumor was calculated by (1) which is generally used from the value measured by vernier caliper.

(1) Tumor  volumemm3=ab2,a: length,  b: width

### 2.6. Tumor Weight Measurement

At Sarcoma, Scc-7, the mouse was sacrificed by cervical dislocation on the 14th day after ECT was performed, the tumor was removed, and the weight was measured. Ehrlich also sacrificed the mouse by cervical dislocation on day 33 after performing ECT, and the tumor was removed, and the weight was measured.

### 2.7. Tumor Inhibition Rate

The tumor inhibition rate was calculated from formula (2) based on the tumor weight.

(2) $$\begin{array}{c} Tumor\;\;inhibition\;\;rate\left(\;\%\;\right)=\frac{\left(Cw\text{-}Tw\right)}{Cw}\times 100 \end{array}$$

where Cw is average tumor weight of the target group and Tw is average tumor weight of specimen group.

### 2.8. Tumor pH Measurement Study

Ehrlich tumor cells were inoculated on the upper part of the right foot of the ICR mouse, and ECT was performed when the major axis and the minor axis of the tumor became about 1cm × 1cm in size. Immediately thereafter, a pH measuring electrode was pierced into the cathode side and the anode side, and the pH value of the control, 10mA × 150secs, 10mA × 30secs, and 10mA × 600secs group was measured five times each.

### 2.9. Review at the Histological Level

Inbred's C3H/HeNCrj mouse was used at Japan SLC. Co., Ltd. In order to familiarize themselves with the breeding conditions of this study, we purchased preliminary breeding for one week and used it for research. Rearing conditions were bred under Conventional conditions.* The room temperature was 22 ± 3*°*C and the humidity was 60 to 70%. In the light control, water and bait (CA-1, Japan CLEA Co., Ltd.) freely ingested the light period of 12 hours from 6:00 A.M. to 6:00 P.M. *

As a comparative control group, the control group with only tumor inoculation and the antitumor effect group were grouped into 4 groups: tumor + 10mA × 15 secs group, tumor + 10mA × 300sec group, tumor + 10mA × 60 sec group, and 10mA × 150sec group. In this study, male mice of 6 weeks of age in each group were used in each group.

The SCC-7 squamous cell carcinoma was inoculated into C3H mice and used for research. Those stored in liquid nitrogen were warmed and defrosted with a sufficiently humidity incubator under 5% carbon dioxide, mixed with Fetal Bovine Serum in RPMI 1640, and kept in a 2- or 3-month incubator. Approximately 1×10^6^ cells were poured from the mixed solution and mixed with 0.05 ml serum-free medium and used for research. The inoculation site was inoculated subcutaneously in the upper right part of the mouse's right foot. Pentobarbital sodium was purchased from Dainippon Pharmaceutical. It was formulated with 0.45% physiological saline to a concentration of 10%, administered intraperitoneally, and used for anesthesia. The dose concentration was 10ml/kg of 10% Pentobarbital sodium. The study was initiated approximately 10 minutes after peritoneal administration and was used during ECT study.

ECT was performed when the major axis and the minor axis of the tumor inoculated on the upper part of the mouse's right foot became 1cm × 1cm in size.

As the treatment condition, ECT was performed under Pentobarbital sodium anesthesia with the electrode injected into the entire tumor so that the distance between the anode and the cathode was within 10mA × 150 secs, and the current used was 10mA.

Wounds were carefully disinfected with 70% ethanol before and after the insertion of the electrode. ECT was performed on the tumor inoculated on the upper part of the mouse's right foot, and the mice were sacrificed by cervical dislocation (cervical vertebral dislocation) 6, 12, and 24 hrs after each group ECT, and the tumor was removed. The excised tumor was fixed in tissue with 10% formalin.

Tissue sections of controls and tumor tissues after each treatment were prepared, and the staining method was carried out by Hematoxylin and Eosin (HE) staining, apoptosis stained apoptosis antibody staining with ApopTag®'s KIT.

### 2.10. Statistical Analysis

All measurements made in this study were shown in average ± SD. SAS was used for statistical analysis. Parametric ANOVA was used to verify differences in the tumor volume and weight and the number of apoptotic cells in each group.* P* values less than 0.05 were considered significant.

This study has been approved by the Suzuka University of Medical Science Animal Research Ethics Committee (Ref: 2003/423/15).

## 3. Result

### 3.1. Study of Cell Level

**Figure 3:**
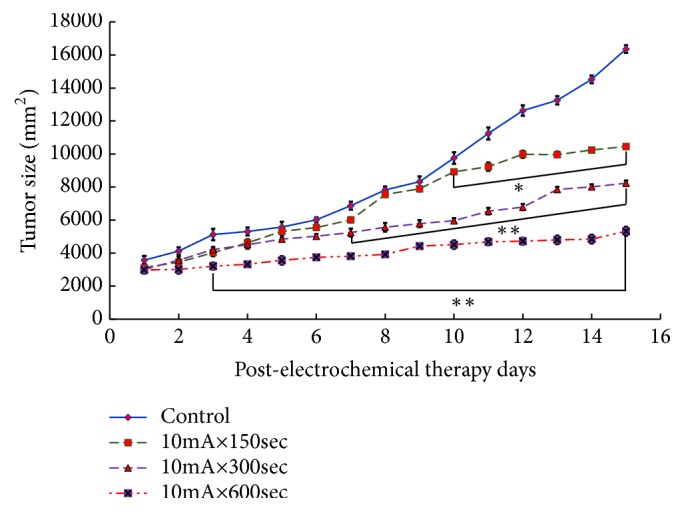
Changes of the tumor volume after Sarcoma-180 inoculation in the groups of control, 10mA×150sec, 10mA×300sec, and 10mA×600sec (*∗ p<0.05*, *∗∗ p<0.01*). The results represent ± S.D. from 8 to 10 mice.

The average value and standard error of the tumor volume obtained by the measurement and the weight of the tumor after the removal and the tumor suppression rate are shown as the mean value and standard error for each tumor. The horizontal axis represents the number of days elapsed after treatment, the vertical axis represents tumor volume, the horizontal axis represents the study group, and the vertical axis represents the tumor weight after excision divided by tumor. In addition, the mean value and standard error of intratumor pH value are shown. The horizontal axis represents the research group, and the vertical axis represents the tumor pH value.  Figure 3 shows the tumor inhibition rate by Sarcoma-180 cells. A statistically significant difference was observed from the 10mA × 150sec group as compared with the control group (*p<0.05*). A statistically significant difference was observed in the 10mA × 300sec group and the 10mA × 600sec group as compared with the control group (*p<0.05, P<0.01*).

**Figure 4:**
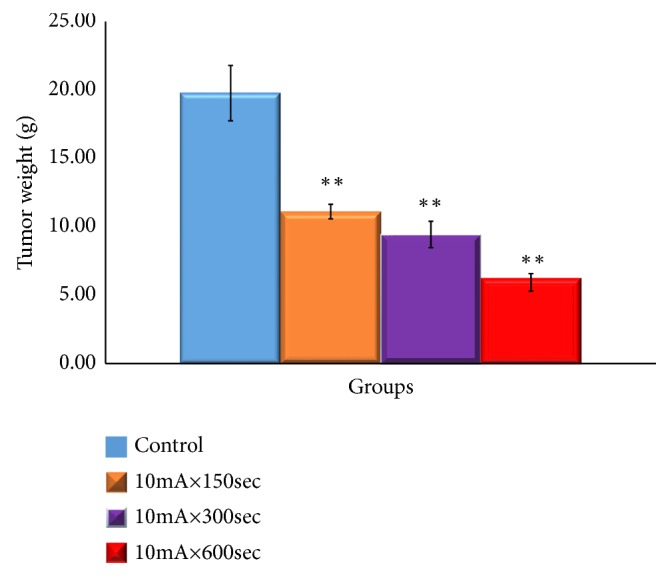
Tumor growth-inhibitory effect of ECT in Sarcoma-180 cells-bearing mice. Changes in the weight of the tumor. After tumor implantation, we measured the weight of the fifth week of the tumor of each group. The results represent ± S.D from 8 to 10 mice. *∗∗* Significantly different from the control group (*p <0.01*).

The tumor weight by Sarcoma-180 cells is shown in Figure 4. A statistically significant difference was observed in the 10mA × 150sec group, 10mA × 300sec group, and 10mA × 600sec group as compared with the control group (*P <0.01*).

**Figure 5:**
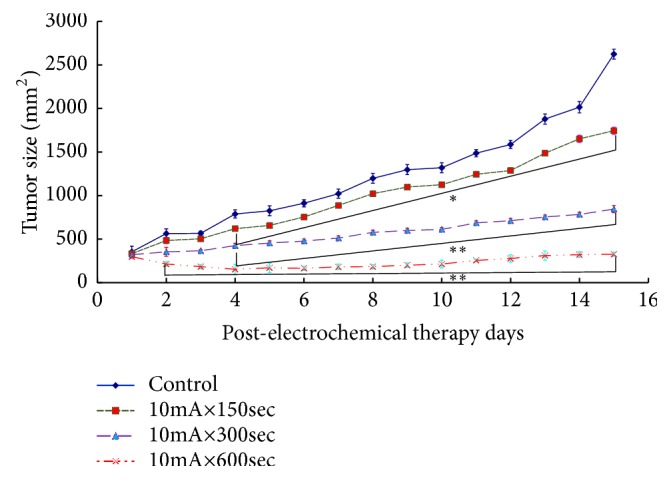
Changes of the tumor volume after SCC-7 inoculation in the groups of control, 10mA×150sec, 10mA×300sec, and 10mA×600sec (*∗ p<0.05*, *∗∗ p<0.01*). The results represent ± S.D. from 8 to 10 mice.

Figure 5 shows the tumor inhibition rate by Scc-7 cells. A statistically significant difference was observed from the 10mA × 15 sec group as compared with the control group (*p <0.05*).

A statistically significant difference was observed in the 10mA × 300sec group and the 10mA × 600sec group as compared with the control group (*p <0.05, P <0.01*).

**Figure 6:**
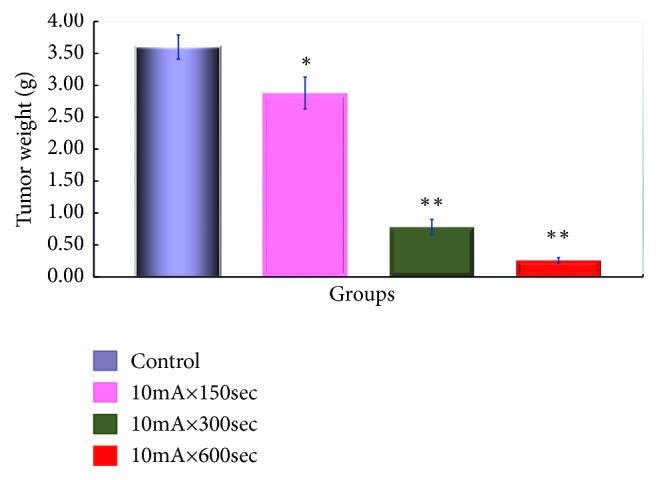
Tumor growth-inhibitory effect of ECT in Sarcoma-180 cells-bearing mice. Changes in the weight of the tumor. After tumor implantation, we measured the weight of the fifth week of the tumor of each group. The results represent ± S.D from 8 to 10 mice. *∗∗* Significantly different from the control group (*p <0.01*).

The tumor weight by Scc-7 cells is shown in Figure 6. A statistically significant difference was observed from the 10mA × 150sec group as compared with the control group (*p <0.05*).

A statistically significant difference was observed in the 10mA × 300sec group and the 10mA × 600sec group as compared with the control group (*p<0.05, P<0.01*). Therefore, all tumor suppressing effects were observed in the ECT treated group.

**Figure 7:**
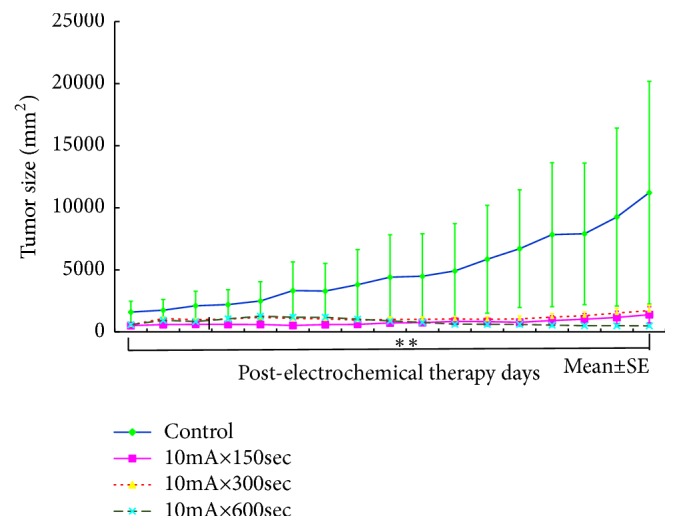
Changes of the tumor volume after Ehrlich carcinoma inoculation in the groups of control, 10mA×150sec, 10mA×300sec, and 10mA×600sec (*∗∗ p<0.01*). The results represent ± S.D. from 8 to 10 mice.

Figure 7 shows the tumor inhibition rate by Ehrlich carcinoma. A statistically significant difference was observed from the 10mA × 150sec group as compared with the control group (*p <0.05*).

A statistically significant difference was observed in the 10mA × 30 sec group and the 10mA × 600sec group as compared with the control group (*p <0.05, P <0.01*).

**Figure 8:**
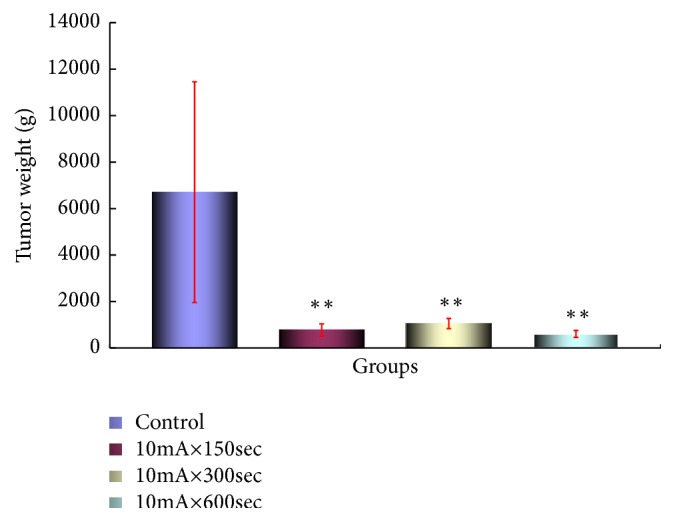
Tumor growth-inhibitory effect of ECT in Ehrlich carcinoma cells-bearing mice. Changes in the weight of the tumor. After tumor implantation, we measured the weight of the fifth week of the tumor of each group. The results represent ± S.D. from 8 to 10 mice. *∗∗* Significantly different from the control group (*p <0.01*).

The tumor weight by Ehrlich carcinoma cells is shown in Figure 8. A statistically significant difference was observed in the 10mA × 150sec group, 10mA × 300sec group, and 10mA × 600sec group as compared with the control group (*P <0.01*). Therefore, all tumor suppressing effects were observed in the ECT treated group.

**Figure 9:**
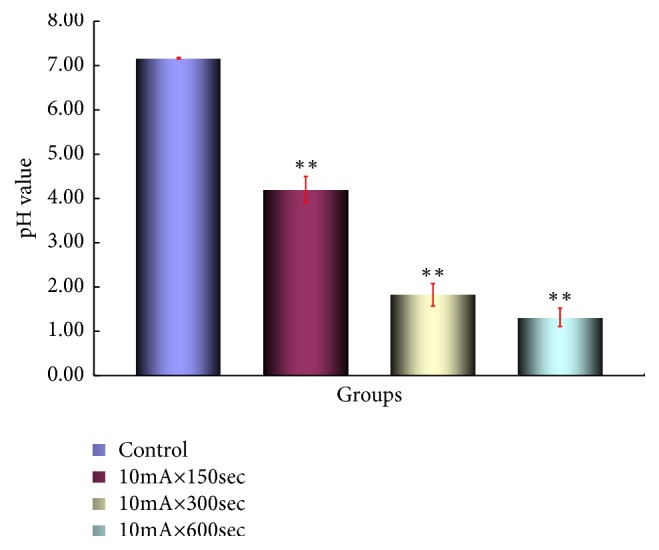
pH in the tumor on the anode side. Each bar represents the mean value SE for 5 points from 8 to 10 mice.. Significantly different *∗p<0.05*, *∗∗p<0.01* control vs. 10mA×150sec, control vs. 10mA×500sec, control vs. 10mA×300sec, 10mA×150sec vs. 10mA×500sec, and 10mA×150sec* vs.* 10mA×300sec.

**Figure 10:**
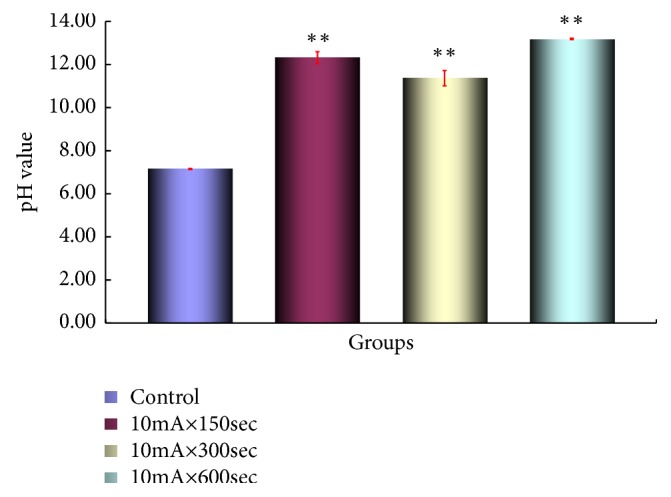
pH in the tumor on the cathode side. Each bar represents the mean value SE for 5 points from 8 to 10 mice. Significantly different *∗p*<*0.05*, *∗∗p<0.01* control* vs.* 10mA×150sec, control* vs.* 10mA×500sec, control* vs*. 10mA×300sec, 10mA×150sec vs. 10mA×500sec, and 10mA×150sec* vs.* 10mA×300sec.

PH measurement on intratumor pH results on the anode side are shown in Figure 9 and pH on the cathode side is shown in Figure 10. A statistically significant difference was observed in the anode group and the cathode group compared to the control group (*p <0.01*).

### 3.2. Review at the Histological Level

**Figure 11:**
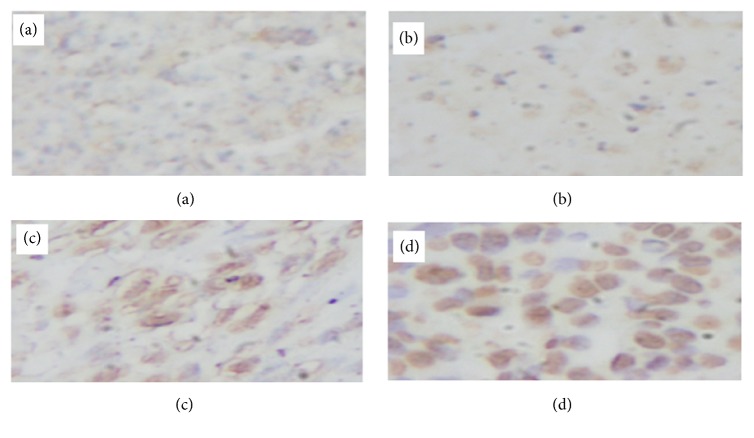
Findings of apoptosis occurring in SCC-7 of C3H mouse after electrochemical therapy. ApopTag® Harris hematoxylin stain. (a) Control group, scattered with few apoptotic cells (x200). (b) 10mA×150sec group with mild degree of apoptosis 6 hours after electrochemical therapy (x400). (c) 10mA×300sec group with moderate degree in apoptosis 6 hours after electrochemical therapy (x400). (d) 10mA×600sec group showing marked apoptosis 6 hours after electrochemical therapy (x400). The distribution of apoptosis of the tumor. Quantitation was performed as described in the text and the distribution of apoptotic bodies expressed in relation to position on the tumor cells.

**Figure 12:**
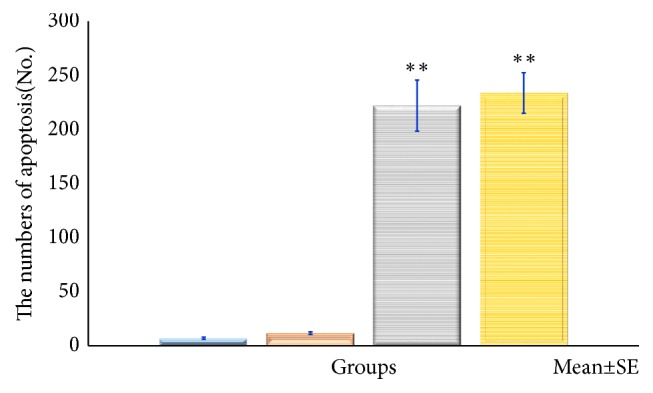
The number of cells undergoing apoptosis after electrochemical therapy in 6hr after treatment. The distribution of apoptosis of the tumor. Quantitation was performed as described in the text and the distribution of apoptotic bodies expressed in relation to position on the tumor cells. Significantly different *∗∗p<0.01*, control* vs*. 10mA×150sec, 10mA×300sec, and 10mA×600sec. The results represent ± S.D. from 8 to 10 mice.

**Figure 13:**
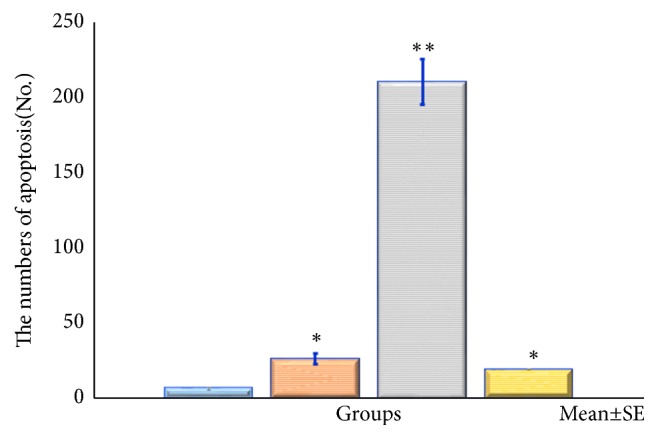
The number of cells undergoing apoptosis after electrochemical therapy in 12hr after treatment. The distribution of apoptosis of the tumor. Quantitation was performed as described in the text and the distribution of apoptotic bodies expressed in relation to position on the tumor cells. Significantly different *∗p<0.05*, *∗∗p<0.01*, control* vs*. 10mA×150sec, 10mA×300sec, 10mA×600sec. The results represent ± S.D. from 8 to 10 mice.

**Figure 14:**
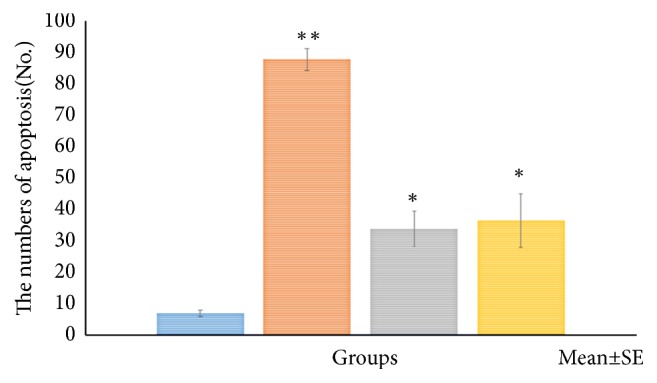
The number of cells undergoing apoptosis after electrochemical therapy in 24hr after treatment. The distribution of apoptosis of the tumor. Quantitation was performed as described in the text and the distribution of apoptotic bodies expressed in relation to position on the tumor cells. Significantly different *∗p<0.05*, *∗∗p<0.01*, control* vs.* 10mA×150sec, 10mA×300sec, and 10mA×600sec. The results represent ± S.D. from 8 to 10 mice.

*HE Staining and Apoptosis Antibody Staining*. Figures 11–14 show the changes in the number of apoptosis in each group with microscopic observation of slides stained with apoptosis antibody at a magnification of 400 times. For the observation of apoptosis 6 hours after ECT treatment, a statistically significant difference was observed in the 10mA ×300sec group and the 10mA × 600sec group as compared with the control group (*p <0.01*). 12 hours after ECT treatment observation of apoptosis showed* (p <0.05*) in the 10mA × 150sec group and the 10mA × 600sec group as compared with the control group, and a statistically significant difference in the 10 mA × 600 sec group (*p <0.01*) was admitted.

For the observation of apoptosis 24 hours after ECT treatment, there was a statistically significant difference in the 10mA × 150sec group (*p <0.01*), the 10mA × 300sec group, and the 10mA × 600sec group (*p <0.05*) as compared with the control group.

**Figure 15:**
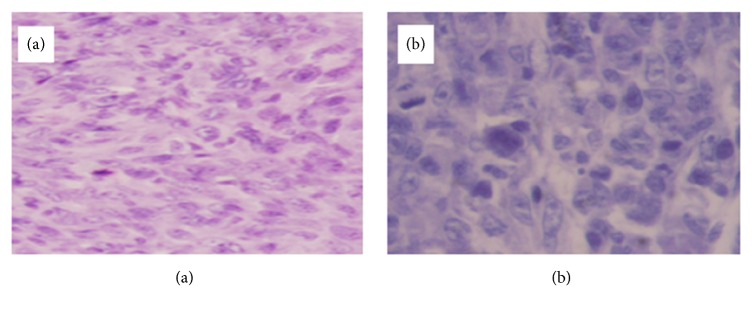
Microscopic finding of squamous cell carcinoma (1x1 cm) occurring on the right upper thigh after inoculation of SCC-7 in control group (a) and 10mA×600sec group (b). (a) There are mainly well-differentiated spindle cells with whirled solid pattern and some mitotic figures (H & E, x200). (b) Tissue section taken 6 hours after treatment in experimental group received 1 coulomb. There are fully differentiated spindle cells without difference comparing control (H & E, x400).

**Figure 16:**
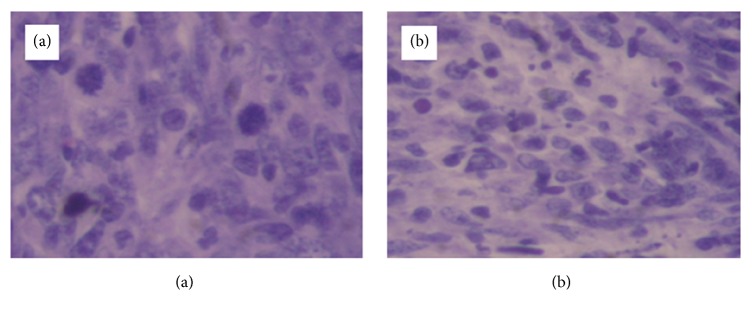
Microscopic finding of squamous cell carcinoma (1cm x 1cm) occurring on the right upper thigh after inoculation of SCC-7 in 10mA×600sec group. (a) Tissue section taken 6 hours after treatment. There is focal cellular apoptotic body of SCC (H & E, x400). (b) Tissue section taken 24 hours after treatment. There is destructive change including moderately fragmented and necrotic neoplastic cells (H & E, x400).

**Figure 17:**
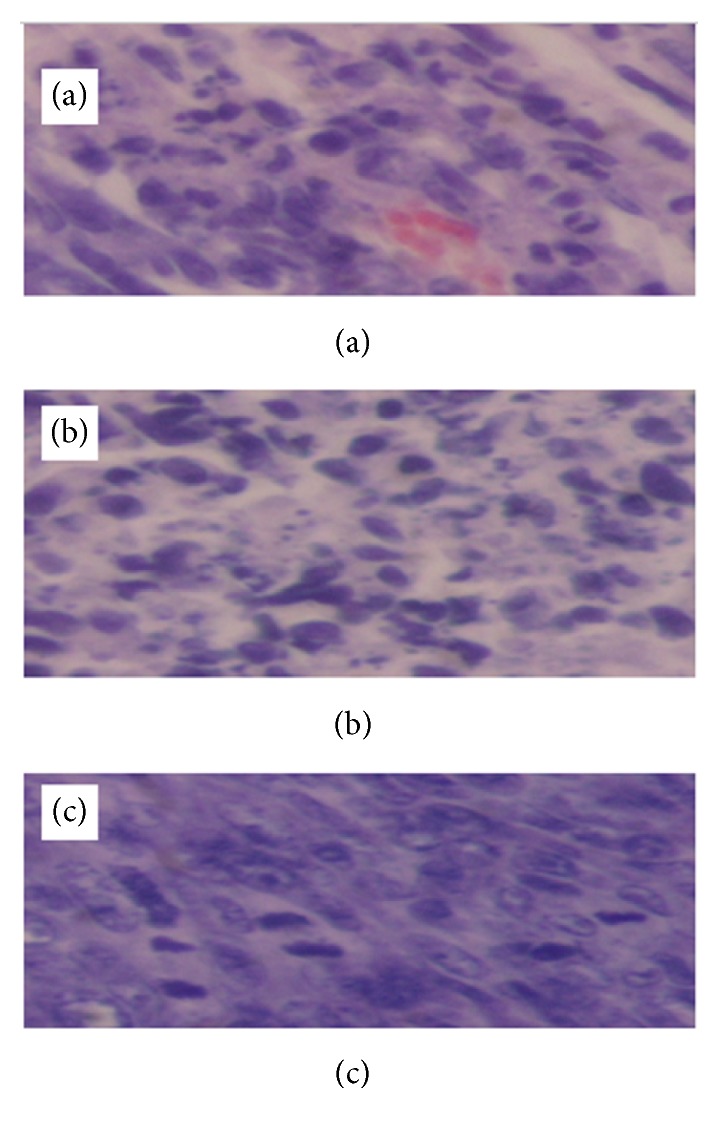
Microscopic finding of squamous cell carcinoma (1x1 cm) occurring on the right upper thigh after inoculation of SCC-7 in 10mA×600sec group. (a) Tissue section taken 6 hours after treatment. There are many pyknosis neoplastic cells and also some typical spindle cells with mitosis (H & E, x400). (b) Tissue section taken 12 hours after treatment. There are many destructive neoplastic cells (H & E, x400). (c) Tissue section taken 24 hours after treatment. There are diffuse, numerous cellular debris derived from neoplastic cells (H & E, x400).

**Figure 18:**
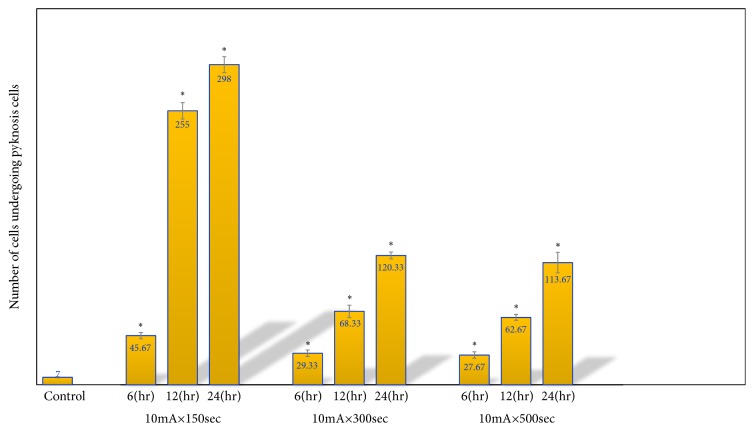
Microscopic finding of squamous cell carcinoma (1x1 cm) occurring on the right upper thigh after inoculation of SCC-7 in 10mA×150sec group, 10mA×300sec group, and 10mA×600sec group. Tissue section taken 6, 12, and 24 hours after treatment. There are many pyknosis neoplastic cells and also some typical spindle cells with mitosis (H & E, x400). (*∗ p<0.05*). The results represent ± S.D. from 8 to 10 mice.

Slides of HE staining are shown for each group in Figures 15–17, and pyknosis neoplastic cells are shown in Figure 18.

A statistically significant difference was observed in the 10mA × 150sec group, the 10mA × 300sec, and the 10mA × 600sec group as compared with thec Control group (*p <0.01*).

Slides of HE staining are shown for each group in Figures 15–17, and pyknosis neoplastic cells are shown. Statistically significant difference was observed in the 10mA × 150sec group, 10mA × 300sec group, and 10mA × 600sec group (*p <0.01*) compared with the control group.

## 4. Discussion 

In 1978 Nordenstroem got antitumor effect against lung metastatic malignancy and lung cancer by electrification therapy. Since then, it began to gather attention as a treatment for tumors [5]. Adaptation conditions are solid tumors and are attempted for tumors of all tissue type. In particular, the results of CR + PR exceeding 90% have been reported in skin cancer, malignant melanoma, superficial squamous carcinoma, vulva cancer, and scar cancer [9]. However, there are still many theories about its principle, but none have been confirmed.

It appears that ECT caused rapid and polar-dependent changes in physiological solutions in cancer tissues, and similar electrochemical processes mediated a decrease in observed tumor growth. ECT is potentially useful as an adjuvant therapy because it reduces the local tumor burden but does not alter the extent of metastasis [5].

According to Xin et al.'s research, the anode was inserted into the tumor in lung cancer, and the cathode was inserted from the end of the tumor at least at the distance of the diameter of the tumor, and ECT was performed. As a result, I discovered that it was damaged by the hydration that occurred [10]. From this, as moisture moves from the anode to the cathode by performing ECT, local hydration around the cathode dehydrates around the anode. That is, it is suggested that both the cathode and the anode are effective.

In addition, since the dehydration effect occurs on the anode side, the electrical resistance rises, and the temperature of the tissue rises, so it seems that hyperthermia effect may be present.

However, it has been reported that findings by hyperthermia were not observed in local temperature measurements during the ECT performed by Miklavcic et al. [11].

Also, in this study, when observing the slide of HE staining, hyperthermia effect was not observed, so it is considered that there is no hyperthermia effect in ECT. It has also been reported that treatment with low current for a long time gave better results than treatment with high current for a short time [10].

Even in this study, ECT effect was observed in the 10mA × 300sec group and 10mA × 60 sec group, but in the 10mA × 150sec group, the effect of ECT was not appreciated so much, similar to the report of Xin et al. The amount and treatment time are considered to be important factors in ECT.

According to Ito et al.'s research, there is a report that confirmed that 5-fluorouracil concentration was remarkably higher in the tumor tissues of the ECT group than in the control group.

This is thought to be the result of anticancer drug being taken into cells of the tumor tissue by opening the Na^−^ and K^+^ channel by ECT. Protein degeneration also occurs by performing ECT.

Studies using Li et al. reported that radioimmunoassay markedly decreased albumin and globulin around the cathode and anode in dog liver. According to Ito et al.'s research, hemoglobin is converted into iron (III) protoporphyrin chloride (C_34_H_32_ClFIIIN_4_O_4_) = chlorohemin which is acidic Hemin on the anode side and iron (III) protoporphyrin (C_34_H_32_O_4_N_4_FeOH)) = Hematin (hematin) has also been reported to occur [12].

A research report has been made that in the in vitro study using Yun et al.'s Human KB Cells, the pH is 4.53 around the anode and 10.46 around the cathode and the pH returns to 7.5 after 24 hours [13].

In the* in vivo* study using mouse Scc-7 cells in this study, the intratumoral pH value was 4.20 at 10mA × 150secs and 1.82 at 10mA × 300secs around the anode and 10mA × 600secs.

It was confirmed that the pH value was changed to strongly acidic with 1.31 at the periphery of the cathode, 12.31 at 10mA × 150secs, 11.37 at 10mA × 300secs, and 13.15 at 10mA × 600secs.

Since this result is similar to that reported by Yun et al., ECT was found to have a therapeutic effect due to pH change. Changes in intracellular pH are indicated in the same way as changes in extracellular pH; for example, H^+^ increases and as the extracellular pH decreases, intracellular pH decreases. Because it is strongly alkaline around the cathode, it causes liquefaction necrosis and the activity of the cell is quickly lost because it is strongly acidic around the anode. In addition, the intracellular alkalization increases the flow of calcium, resulting in an increase in intracellular Ca^2+^. Since Ca^2+^ in electrolyte is a determinant in toxicological processes in various pathologies, cellular disorder occurs when the intracellular Ca_2_^+^ stability breaks down. When Ca^2+^ becomes excessive, it is thought that Ca^2+^ plays an important role of killing the cell, since it can cause cell death.

Changes in the intracellular pH and the shape of the ions in the cell are thought to cause apoptosis [14–16].

In 1995, a study using Yoshida sarcoma cells in Ito et al.'d Dynasty Rat also showed a faint ladder pattern by DNA agarose gel electrophoresis by Wyllie's method and a nucleus by nick-end labeling method. The positive staining of the stain is recognized [17].

In this study, apoptosis antibody staining was performed instead of agarose gel electrophoresis or nick-end labeling method; it was found that after hrs it changed to 10mA × 300secs and 10mA × 600secs group, after 1hr it changed to 10mA × 300secs group, and after 24hrs there was a significant increase in apoptosis in each group compared to the control group. In addition, occurrence of apoptosis was confirmed particularly in the vicinity where the electrode was inserted.

This result is similar to Ito et al.'s, and it is certain that ECT causes apoptosis. This result suggested that the effect of apoptosis on tumor treatment is present in ECT. From this result, when the condition for treating ECT is as low as 10mA × 150secs, the time at which apoptosis occurs depending on the condition to treat at 10 mA × 300 secs, 10 mA × 300 secs, when the condition is as high as 10mA × 600secs, after 6hrs, after 12hrs, and after 24hrs, it seems that there is a difference between them.

Also, by performing ECT, high concentration of Na^+^, K^+^ is confirmed around the cathode, and high concentration of Cl^−^ around the anode has been confirmed. Other ions Ca^2+^, Mg^2+^, Mn^2+^, Cu^2+^, and A^l3+^ also for them to integrate moving speed inversely proportional to the magnitude of the diameter of the atoms, Na^+^, later compared to K^+^, for the movement difference Na^+^, K^+^ more accumulates around the cathode. Nerve excitability occurs due to more Na^+^ and K^+^ gathered. When cells are subjected to appropriate physicochemical stimuli, the ion permeability of the cell membrane changes rapidly, resulting in stimulation and changes.

The generation of this action potential is called excitation, and the cell membrane having the excitability, and the excitability by exciting property according to the stimulation is called an excitable membrane. Although it has nothing to do with this study directly, it is certain that such a thing also causes some changes in the tumor as it is caused by ECT.

Radicals oxidized by Cl_2_, O_2_, and O_3_ are adsorbed on the anode [18], which in turn certainly causes necrosis [19]. In the vicinity of the cathode, oxygen causes necrosis to be expelled from the tissue by hydrogen. Even in this study, it was confirmed that necrosis was caused by HE staining of tissues, so ECT caused necrosis. As described above, it is considered that ECT principle is to change the electrolyte concentration of the cytoplasmic substrate by changing the intracellular pH, thereby inducing cell damage and cell death.

In this study, the tumor suppressing effect of Scc-7 was observed in the 10mA × 300sec group and the 10mA × 600sec group.

This is similar to the study by Yun et al. By passing electricity through, pH of the tumor is attracted on the anode side, Cl_2_ is generated by binding binds H_2_ O in the tissue, Cl_2_ generated, and HCl, HClO, Na^+^ attracted to OH^−^ on the cathode side changed to strongly alkaline by NaOH generated, which is thought to be the result of affecting tumor cells [20].

Necrosis occurred within the range where the electrochemical reaction by ECT was prevailing, and because it was reabsorbed, a difference was observed in the tumor weight. This is thought to be based on the same principle as reported by Ashok K. Vijh et al. [19]. As a result, it was suggested that tumor suppressing effect was observed in ECT in Sarcoma-180 as tumor suppressing effect was observed in each group than tumor inhibition rate.

In the study using Ehrlich, it is considered that the ascitic tumor appeared more strongly in the electrical response as the reason why clear tumor suppressing effect was observed in each group in the tumor growth measurement. Also, necrosis occurred within the range where the electrochemical reaction by ECT was well-received, and because it was reabsorbed, a difference was observed in the tumor weight. The tumor of Scc-7 was sarcoma and Ehrlich's cancer was ascites. Therefore, the ECT treatment showed a significant decrease in the water content.

In this result as well, it seems that it is based on the same principle as that reported by Ashok et al. and Gu et al. [19, 21]. As a result, tumor suppression effect was observed in each group than tumor inhibition rate. CR was observed in the 10mA × 600sec group. From these results, it was confirmed that ECT has tumor suppressing effect in this study. Moreover, by conducting reresearch in the future, it is considered possible to lead to more reliable research, further development, and use of ECT system. We have great potential for future devices that will enable rapid clinical decision-making. ECT reduces medical expenses and patient stress. However, it is also necessary to verify the reliable effect of cancer treatment for each type of cancer related important issues.

## 5. Conclusion

In this study, a significant increase in apoptosis was confirmed in each ECT group compared to the control group. Treatment effects by ECT were also observed in tumor growth measurement studies and tumor weight measurement studies. From these research results, ECT is considered to be effective as a tumor treatment method.

## Data Availability

The data used to support the findings of this study are available from the corresponding author upon request.
